# Prognostic factors for mortality in age-stratified elderly sepsis patients: A retrospective cohort study based on the MIMIC-IV database

**DOI:** 10.1097/MD.0000000000048209

**Published:** 2026-04-10

**Authors:** Jiao Chen, Jinfeng Lin, Hao Wang, Chunfeng Gu, Xiang Lu, Chongxiu Sun

**Affiliations:** a Collaborative Innovation Center for Cardiovascular Disease Translational Medicine, Nanjing Medical University, Nanjing, China; b Department of Critical Care Medicine, Sir Run Run Hospital, Nanjing Medical University, Nanjing, China; c Department of Critical Care Medicine, Affiliated Nantong Hospital 3 of Nantong University, Nantong Third People`s Hospital, Nantong, China; d Copper Ridge Health Care, West Jordan, Utah; e Ctrip Infrastructure Service, Trip.com Group Ltd, Nantong, China; f Department of Geriatrics, Sir Run Run Hospital, Nanjing Medical University, Nanjing, China.

**Keywords:** acute kidney injury, age stratification, elderly, mortality, prognostic factors, sepsis

## Abstract

Sepsis represents a major cause of death among elderly patients; however, prognostic factors specific to different age strata within this population remain insufficiently characterized. This retrospective cohort study aimed to identify early predictors of mortality in elderly sepsis patients using the MIMIC-IV database. We included 13,461 sepsis patients aged ≥60 years, stratified into younger-old (60–74 years; n = 7618) and older-old (75–90 years; n = 5843) groups. Propensity score matching was applied to balance baseline characteristics, yielding 4657 matched patients per group. Multivariable logistic regression, receiver operating characteristic analysis, and Kaplan–Meier survival curves were employed to assess clinical and biochemical markers for 28-day mortality and to identify age-specific prognostic indicators. These findings underscore the importance of age-stratified prognostic models to guide targeted clinical interventions and potentially improve outcomes in elderly patients with sepsis. Lactate dehydrogenase (LDH) and the PaO_2_/FiO_2_ ratio emerged as key mortality predictors in the younger-old group, whereas urea nitrogen and hypoalbuminemia were predominant in the older-old group. Urine output served as a common predictor across both strata, with oliguria associated with a 2.5-fold increase in mortality risk among older patients. While a combination of predictors enhanced prognostic accuracy in younger-old patients, this was not observed in the older-old group, suggesting a greater influence of frailty and comorbidities in advanced age.

## 1. Introduction

Sepsis, defined as life-threatening organ dysfunction caused by a dysregulated host response to infection, is a major burden on healthcare systems and a critical global health issue. In 2017, there were an estimated 48.9 million cases of sepsis and 11 million sepsis-related deaths worldwide, accounting for approximately 20% of all global deaths.^[1]^ With shifting demographics, older adults are now constituting an increasing proportion of critically ill patients. Age-related factors, such as multiple chronic conditions, impaired immune function, malnutrition, and frailty, significantly increase mortality in patients with critical illnesses, such as sepsis. In the United States, sepsis caused 2,01,092 deaths in 2019, with the majority occurring in individuals aged ≥ 65years.^[2]^ Mortality also increases with age, with the sepsis-related death rate rising sharply from 201.1/1,00,000 in those aged 65 to 74 years to 858.3/1,00,000 in those aged ≥85 years.^[3]^

Sepsis typically has a rapid onset and deteriorating course; however, it is treatable. Evidence has consistently demonstrated that early and targeted interventions significantly improve outcomes.^[4]^ Therefore, identifying key prognostic factors in patients is crucial for prioritizing care and implementing timely treatment. Previous studies have primarily focused on specific biomarkers or genes for clinical diagnosis and outcome prediction.^[5-8]^ While these indicators show great promise owing to their high accuracy and specificity, their utility is often limited in clinical practice owing to issues of accessibility and standardization. Furthermore, most population-based studies do not differentiate by age, making it difficult to assess how the elderly respond to sepsis due to their unique physiological characteristics.

In contrast, clinical indicators and basic biochemical markers, which are readily available, cost-effective, and immediately applicable, are more commonly used in sepsis management.^[9]^ The elderly population exhibits distinct biological and clinical characteristics that influence sepsis outcomes. This finding highlights the need for age-stratified analyses to better understand the prognosis of this vulnerable group. Unfortunately, reliable early prognostic indicators specifically designed for older adults are underexplored in the current literature.

This study aimed to bridge this gap by retrospectively analyzing clinical data from the Medical Information Mart for Intensive Care IV(MIMIC-IV) database to investigate the prognostic value of early indicators in elderly patients with sepsis. Specifically, the study stratified patients into 2 groups: younger elderly (aged 60–74 years) and older elderly (aged 75–90 years). We evaluated the relationships between various early indicators and mortality outcomes. Through this analysis, we hope to identify age-specific prognostic markers that could guide early intervention and improve the management of elderly patients with sepsis.

## 2. Materials and methods

### 2.1. Data source

This study is a multi-cohort, retrospective, observational analysis using data from the online international critical care database-Medical Information Mart for Intensive Care IV (MIMIC-IV) v2.0 database. MIMIC-IV is a publicly available Intensive Care Unit (ICU) database that comprises patient-related data collected from the Beth Israel Deaconess Medical Center (Boston) between 2008 and 2019. The original data collection was approved by the Institutional Review Board of Beth Israel Deaconess Medical Center, and the de-identification process for creating the publicly available MIMIC-IV resource was approved by the Institutional Review Board of the Massachusetts Institute of Technology. One of the authors, Jinfeng Lin (Record ID: 48886665]), is a certified user of PhysioNet, having completed the “Protecting Human Research Participants’ exam provided by the National Institutes of Health and signed a data use agreement. This study adhered to the principles outlined in the Declaration of Helsinki. Since the MIMIC-IV database was de-identified in accordance with the HIPAA Safe Harbor standards, the requirement for ethical review and informed consent for this secondary analysis was waived.

### 2.2. Study populations and definitions

All patients aged > 60 years diagnosed with sepsis were eligible for the study. Sepsis was defined according to The Third International Consensus Definitions for Sepsis and Septic Shock (Sepsis-3.0) as the patients had a suspected infection and a Sequential Organ Failure Assessment score ≥2. Exclusion criteria included patients younger than 60 years or older than 90 years, as well as patients whose length of hospital stay was <72 hours. Patients aged ≥ 90 years were excluded because of significant individual variability and shorter life expectancy. Additionally, patients with a history of chronic renal dysfunction were excluded to eliminate confounding effects of preexisting kidney impairment. Only the first admission was considered in patients with multiple ICU admissions. The included patients were stratified into 2 age groups: younger elderly (60–74 years) and older elderly (75–90 years). The stratification aimed to ensure sufficient sample sizes for each group and allow investigation of age-specific differences in prognosis.

### 2.3. Covariates

Baseline patient information was collected as follows: age, gender, weight, height, Charlson comorbidity index, and length of stay in the ICU. Clinical parameters, such as vital signs at the time of admission, were recorded. Laboratory tests, including complete blood count, comprehensive metabolic panel, and arterial blood gas within the first 24 hours of ICU admission, were also collected.

### 2.4. Outcomes

The primary outcome of interest was 28-day all-cause mortality. Secondary outcomes included the incidence of sepsis-related complication- acute kidney injury (AKI), defined as a serum creatinine (SCr) increase of 2.0 to 2.9 times above baseline or urine output < 0.5 mL/kg/h for more than 12 hours.

### 2.5. Statistical analysis

In this study, for the baseline data of older adults with sepsis in different age groups, we used the Kolmogorov–Smirnov test (K–S test) to evaluate the normality of continuous variables; if the *P*-value was <.05, the variable did not conform to a normal distribution, and a nonparametric test was used for the statistical analysis. Normally distributed continuous variables were expressed as mean ± standard deviation, and the Student *t* test was used to analyze the differences between the 2 groups. Categorical variables were expressed as numbers (percentages), and the 2-sided χ^2^ test was used to analyze the differences between the 2 groups. To balance the important characteristics of patients in each group and reduce selection bias, we used propensity score matching and set the caliper value to 0.01. In the prognostic analyses of the overall and subgroups, we used logistic regression analyses to determine the prognostic risk factors. Candidate predictors were selected from baseline and early (first 24 hours) clinical/laboratory variables. Univariate logistic regression was performed first, and variables with *P* < .05 were entered into the multivariable model. Variables with .05 ≤ *P* < .10 were also considered if clinically relevant.

And combined the coefficients of the predictors to create joint predictors. Composite predictors were calculated as a weighted linear combination of selected variables using the multivariable logistic regression coefficients (β) within each age group and normalized by the LDH coefficient: Combination = (Σβ*ᵢ*X*ᵢ*)/β_LDH_. The resulting composite score was also used as the input variable for ROC analyses.

Receiver operating characteristic (ROC) curves were used to assess the differential diagnostic ability of each predictor. They were also grouped according to the cutoff value of the factor with the strongest differential diagnostic ability, and survival analyses were performed using the Kaplan–Meier method.

Missing values were not imputed. Descriptive statistics were calculated using available (non-missing) data, and regression/ROC analyses were conducted using complete cases for the variables included in each analysis. Differences with *P* < .05 were defined as statistically significant. All analyses were performed using SPSS version 26.0, STATA version 12.0, and R version 4.0.2 (Vienna, Austria).

## 3. Results

### 3.1. The baseline information and clinical characteristics

**Figure 1.**
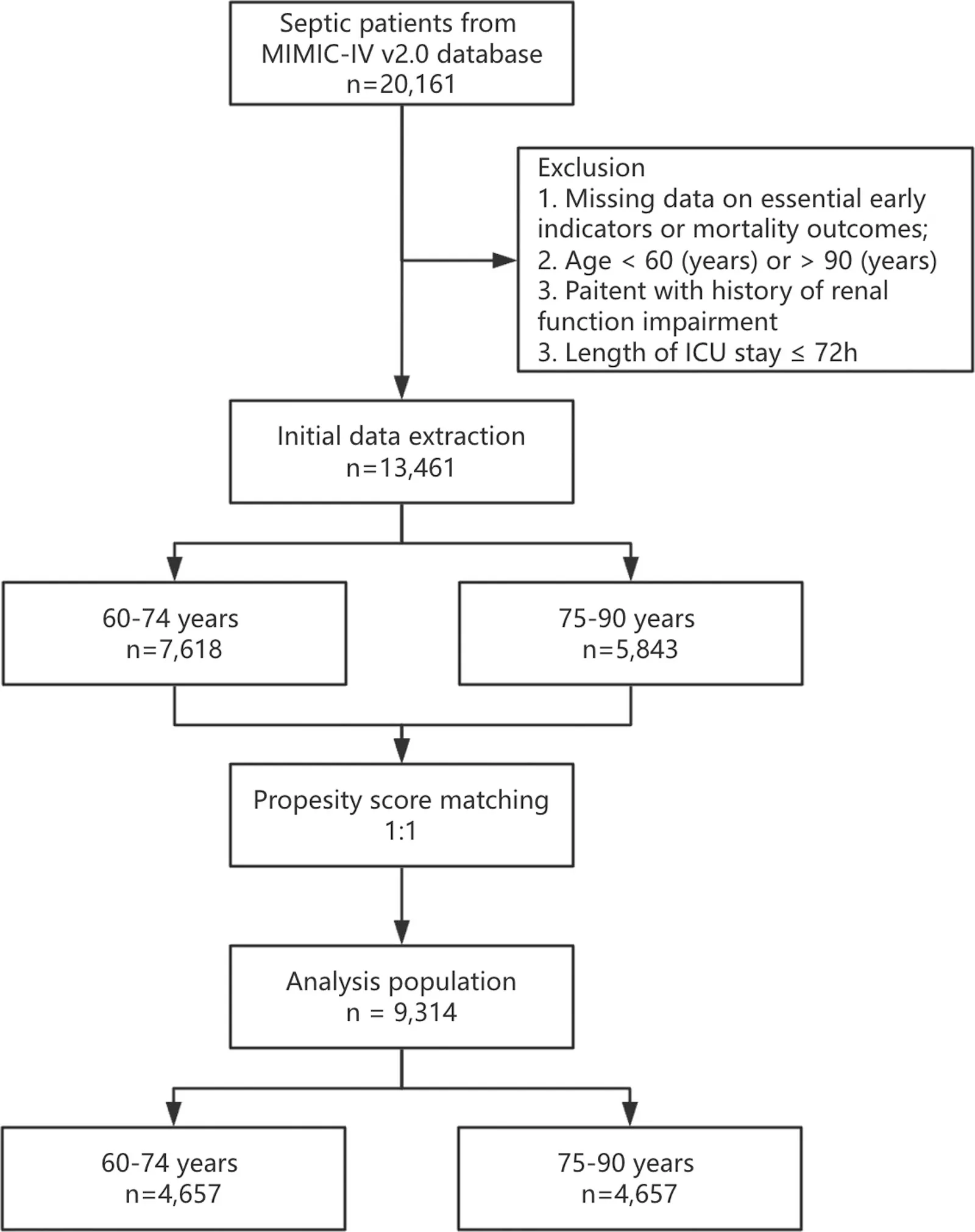
Flowchart of study cohort. MIMIC-IV = Medical Information Mart for Intensive Care IV, ICU = intensive care unit.

In the original dataset, 13,461 patients aged ≥ 60 years with confirmed sepsis were identified, including 7618 patients aged 60 to 74 and 5843 patients aged 75 to 90. After propensity score matching, 9314 patients were retained, with 4657 patients in the 60 to 74 and 75 to 90 age groups. The data collection process and results are shown in Fig.1.

The differences in characteristics between the younger and older groups are summarized in Table 1. The younger elderly group had a significantly greater height and a higher proportion of males than the older elderly group (*P* < .001). Additionally, the younger elderly group had higher maximum levels of urea nitrogen, creatinine, and lactate dehydrogenase (LDH), as well as lower minimum values of pO_2_, PaO_2_/FiO_2_(P/F ratio), and albumin. The younger elderly group also exhibited higher maximum levels of alanine aminotransferase (ALT), total bilirubin, and potassium, and lower minimum values for pH and hemoglobin than the older elderly group. In contrast, the older elderly group had higher maximum sodium levels and a significantly higher 28-day mortality rate than those in the younger elderly group. However, there was no significant difference in the incidence of AKI between the 2 groups.

**Table 1:** The baseline information and clinical characteristics.

Variables	Age group		*P*-value
	60–74	75–90	
n	4657	4657	
Height (cm)	169.55 ± 10.63	167.12 ± 10.64	<.001
Gender			
Male, n(%)	2690 (57.76%)	2466 (52.95%)	<.001
female, n(%)	1967 (42.24%)	2191 (47.05%)	
heart rate, /min	106.51 ± 21.85	106.19 ± 21.94	.406
MBP, mm Hg	54.55 ± 12.72	54.28 ± 12.51	.079
RR, /min	29.22 ± 6.71	29.08 ± 6.54	.440
Temperature,°C	37.47 ± 0.82	37.46 ± 0.74	.487
SpO_2_, %	91.06 ± 6.82	90.96 ± 6.80	.414
Charlson comorbidity index	6.77 ± 2.55	6.82 ± 2.23	.510
LDH, U/L	3.08 ± 2.83	2.78 ± 2.25	<.001
Troponinit, ng/mL	0.82 ± 2.56	0.71 ± 2.34	.236
Creatine kinase isoenzyme, U/L	20.29 ± 53.65	18.01 ± 44.73	.172
Urea nitrogen, mg/dL	37.39 ± 27.99	35.89 ± 25.13	.006
Creatinine,mg/dL	2.13 ± 2.35	1.68 ± 1.41	<.001
Urine output, mL	1667.85 ± 1201.22	1474.87 ± 1004.47	<.001
PaO_2_, mm Hg	99.3 ± 54.65	104.83 ± 64.14	<.001
PaCO_2_, mm Hg	48.22 ± 16.03	47.58 ± 16.28	.117
P/F ratio	204.1 ± 107.97	215.6 ± 126.76	<.001
PT, s	19.01 ± 12.56	18.96 ± 12.85	.845
INR	1.75 ± 1.24	1.75 ± 1.27	.969
Albumin, g/L	2.89 ± 0.68	2.96 ± 0.62	<.001
ALT,U/L	175.99 ± 675.43	115.19 ± 362.14	<.001
Bilirubin total, mg/dL	2.36 ± 5.01	1.36 ± 2.47	<.001
pH	7.31 ± 0.12	7.33 ± 0.1	<.001
Sodium, mEq/L	139.41 ± 5.31	140.27 ± 5.52	<.001
Potassium, mEq/L	4.63 ± 0.82	4.51 ± 0.81	<.001
WBC	15.34 ± 12.01	15.39 ± 13.96	0.838
Hemoglobin, g/dL	9.19 ± 2.11	9.55 ± 2.08	<.001
LOS, d	7.34 ± 7.12	6.81 ± 6.48	<.001
28-d Mortality (n, %)			<.001
0	3634 (78.03%)	3426 (73.57%)	
1	1023 (21.97%)	1231 (26.43%)	
AKI stage			.003
0	342 (11.50%)	327 (10.15%)	
1	443 (14.89%)	493 (15.30%)	
2	1281 (43.06%)	1521 (47.19%)	
3	909 (30.55%)	882 (27.37%)	
Sepsis AKI			.396
0	785 (26.39%)	820 (25.44%)	
1	2190 (73.61%)	2403 (74.56%)	

ALT = alanine aminotransferase, AKI = acute kidney injury, ICU = intensive care unit, INR = international normalized ratio, LDH = lactate dehydrogenase, LOS = length of stay, MBP = mean blood pressure, PaO_2_ = partial pressure of oxygen, PaCO_2_ = partial pressure of carbon dioxide, P/F ratio = PaO_2_/FiO_2_ ratio, pH = potential of hydrogen, PT = prothrombin time, RR = resperatory rate, SpO_2_ = peripheral oxygen saturation,WBC = white blood cell count.

### 3.2. The stepwise logistic regression model

To further investigate the associations between the variables that showed significant differences across different age groups (as detailed in Table 1) and the outcomes of mortality and sepsis-associated AKI, we conducted logistic regression analyses for each age group. This study aimed to assess the potential impact of age on these associations. The results of the stepwise logistic regression model are presented in Tables 2 and 3.

**Table 2:** The results of logistics regression between different age groups, differentially distributed variables and prognostic outcomes of death.

Variables	OR	SE	95% CI low	95% CI high	*P*-value
Age 60–90					
Height	0.99	0.01	0.97	1.01	.278
Gender	0.90	0.18	0.61	1.33	.610
LDH, U/L	1.10	0.03	1.05	1.16	<.001
Urea nitrogen	1.02	0.00	1.01	1.02	<.001
Creatinine	0.88	0.05	0.78	0.99	.035
Urine output	1.00	0.00	1.00	1.00	<.001
PaO_2_	1.00	0.00	1.00	1.01	.461
P/F ratio	1.00	0.00	1.00	1.00	.081
Albumin	0.83	0.09	0.67	1.03	.097
ALT	1.00	0.00	1.00	1.00	.113
Bilirubin total	1.03	0.02	0.99	1.06	.119
pH	3.09	2.26	0.73	13.01	.125
Sodium	0.99	0.01	0.97	1.02	.488
Potassium	1.17	0.11	0.98	1.40	.086
Hemoglobin	1.08	0.04	1.01	1.16	.017
Age 60–74					
Height	0.98	0.01	0.96	1.01	.166
Gender	0.83	0.22	0.49	1.40	.478
LDH	1.08	0.04	1.01	1.16	.018
Urea nitrogen	1.01	0.00	1.00	1.02	.005
Creatinine	0.86	0.07	0.74	1.00	.045
Urine output	1.00	0.00	1.00	1.00	<.001
PaO_2_	1.00	0.00	1.00	1.01	.271
P/F ratio	1.00	0.00	0.99	1.00	.006
Albumin	0.95	0.13	0.72	1.24	.69
ALT	1.00	0.00	1.00	1.00	.151
Bilirubin total	1.03	0.02	0.98	1.07	.223
pH	4.06	3.83	0.64	25.72	.136
Sodium	0.98	0.02	0.95	1.01	.208
Potassium	1.23	0.15	0.97	1.56	.085
Hemoglobin	1.14	0.06	1.03	1.26	.013
Age 75–90					
Height	1.01	0.01	0.98	1.04	.667
Gender	1.12	0.34	0.61	2.05	.721
LDH	1.13	0.05	1.04	1.23	.003
Urea nitrogen	1.02	0.01	1.01	1.03	.001
Creatinine	0.93	0.10	0.75	1.15	.490
Urine output	1.00	0.00	1.00	1.00	.070
PaO_2_	1.00	0.00	0.99	1.01	.762
P/F ratio	1.00	0.00	1.00	1.00	.911
Albumin	0.70	0.13	0.49	1.00	.048
ALT	1.00	0.00	1.00	1.00	.52
Bilirubin total	1.04	0.04	0.97	1.12	.229
pH	1.63	2.03	0.14	18.89	.698
Sodium	1.00	0.02	0.96	1.05	.878
Potassium	1.07	0.16	0.81	1.43	.624
Hemoglobin	1.14	0.06	1.03	1.26	.013

ALT = alanine aminotransferase, CI= confidence interval, LDH = lactate dehydrogenase, OR = odds ratio, P/F ratio = PaO_2_/FiO_2_ ratio, PaO_2_ = partial pressure of oxygen, pH = potential of hydrogen, SE = standard error.

**Table 3:** The results of logistics regression between different age groups, differentially distributed variables and prognostic outcomes of sepsis AKI.

Variables	OR	SE	95% CI low	95% CI high	*P*-value
Age 60–90					
Height	1.02	0.02	0.99	1.05	.113
Gender	1.27	0.39	0.69	2.31	.443
LDH	1.03	0.05	0.94	1.14	.524
Urea nitrogen	1.01	0.01	0.99	1.02	.353
Creatinine	1.03	0.15	0.77	1.37	.838
Urine output	1.00	0.00	1.00	1.00	<.001
PaO_2_, mm Hg	1.00	0.00	0.99	1.01	.988
P/F ratio	1.00	0.00	0.99	1.00	.047
Albumin	0.93	0.16	0.66	1.31	.671
ALT	1.00	0.00	1.00	1.00	.550
Bilirubin total	1.11	0.07	0.99	1.26	0.089
pH	0.20	0.25	0.02	2.31	.196
Sodium	0.95	0.02	0.91	1.00	.040
Potassium	1.20	0.20	0.87	1.67	.274
Hemoglobin	1.10	0.06	0.99	1.23	.082
Age 60–74					
Height	1.01	0.02	0.98	1.05	.467
Gender	1.29	0.52	0.58	2.85	.528
LDH	1.01	0.07	0.88	1.16	.904
Urea nitrogen	0.99	0.01	0.97	1.01	.379
Creatinine	1.73	0.53	0.95	3.16	.073
Urine output	1.00	0.00	1.00	1.00	<.001
PaO_2_	1.00	0.00	0.99	1.01	.806
P/F ratio	1.00	0.00	0.99	1.00	.021
Albumin	0.82	0.19	0.52	1.29	.396
ALT	1.00	0.00	1.00	1.00	.540
Bilirubin total	1.16	0.09	1.00	1.36	.058
pH	1.46	2.32	0.06	32.96	.812
Sodium	0.98	0.03	0.92	1.04	.438
Potassium	1.33	0.32	0.83	2.15	.241
Hemoglobin	1.11	0.08	0.96	1.28	.158
Age 75–90					
Height	1.05	0.03	1.00	1.10	.065
Gender	1.40	0.69	0.54	3.66	.487
LDH	1.04	0.08	0.90	1.20	.585
Urea nitrogen	1.03	0.01	1.00	1.05	.029
Creatinine	0.76	0.15	0.51	1.12	.167
Urine output	1.00	0.00	1.00	1.00	<.001
PaO_2_	1.00	0.00	0.99	1.01	.838
P/F ratio	1.00	0.00	0.99	1.00	.711
Albumin	1.06	0.31	0.60	1.88	.838
ALT	1.00	0.00	1.00	1.00	.975
Bilirubin total	1.03	0.08	0.88	1.20	.750
pH	0.00	0.01	0.00	0.37	.016
Sodium	0.91	0.03	0.85	0.98	.010
Potassium	1.11	0.27	0.69	1.78	.660
Hemoglobin	1.12	0.10	0.95	1.33	.191

ALT = alanine aminotransferase, AKI = acute kidney injury, CI = confidence interval, LDH = lactate dehydrogenase, P/F ratio = PaO_2_/FiO_2_ ratio, PaO_2_ = partial pressure of oxygen, pH = potential of hydrogen, SE = standard error.

For age 60 to 90 years, significant associations were found for LDH (OR: 1.10, 95% CI: 1.05–1.16, *P* < .001), urea nitrogen (OR: 1.02, 95% CI: 1.01–1.02, *P* < .001), and creatinine (OR: 0.88, 95% CI: 0.78–0.99, *P* = .035). Urine output was strongly associated with both mortality and AKI (*P* < .001). The hemoglobin level was also a significant factor (OR: 1.08, *P* = .017). In contrast, height, gender, albumin level, and other factors were not significantly associated with outcomes.

In age 60 to 75 years group, significant associations were observed for lactate dehydrogenase (OR, 1.08; 95% CI: 1.01–1.16, *P* = .018) and urea nitrogen (OR: 1.01, 95% CI: 1.00–1.02, *P* = .005). Creatinine (OR, 0.86; *P* = .045) and hemoglobin (OR, 1.14; *P* = .013) levels were also associated with outcomes. Additionally, the P/F ratio was significant (OR: 1.00, *P* = .006), suggesting a relationship with respiratory function.

In older patients, age 75 to 90 years, significant associations were observed for lactate dehydrogenase (OR, 1.13; 95% CI: 1.04–1.23, *P* = .003) and urea nitrogen (OR: 1.02, 95% CI: 1.01–1.03, *P* = .001). Albumin showed a significant protective effect (OR: 0.70, *P* = .048), whereas hemoglobin was associated with an increased risk (OR: 1.14, *P* = .013).

In secondary analysis across age groups (60–90, 60–75, and 75–90 years), no significant associations were found for height, sex, bilirubin level, or most biochemical markers in secondary analysis. However, the P/F ratio was significant in patients aged 60 to 75 (OR: 1.00, *P* = .021), while urea nitrogen continued to be a predictor of outcomes in the older age groups. Across all age groups, lactate dehydrogenase, urea nitrogen, and hemoglobin consistently showed associations with both mortality and sepsis-associated AKI. Urine output remained a strong predictor in all the age groups. Differences in the associations between age groups suggest that physiological factors, such as respiratory function and renal markers, may play a more prominent role in older patients.

### 3.3. ROC analysis

**Figure 2.**
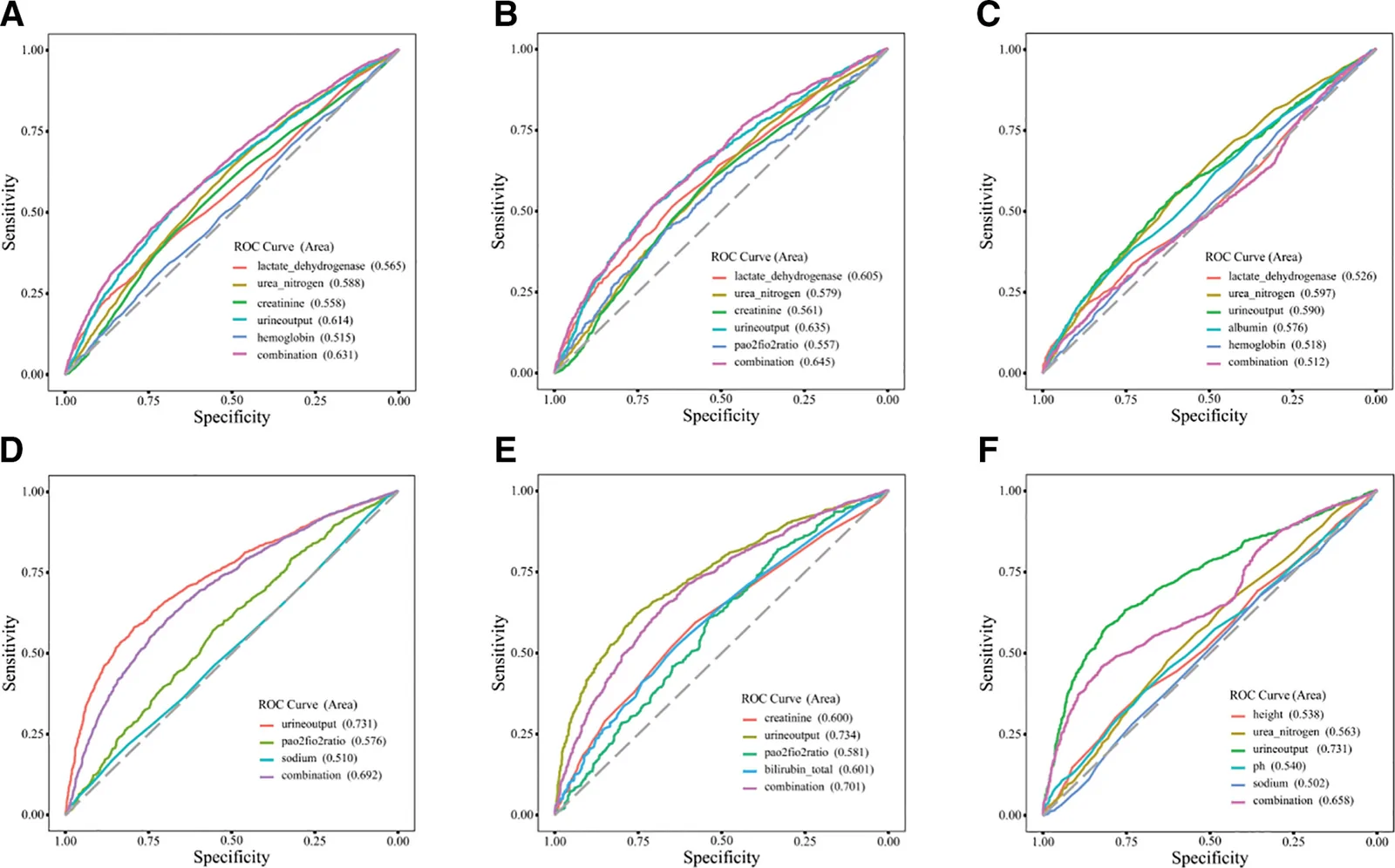
ROC curves evaluating the diagnostic performance of various predictors across different age groups and outcomes. (A) Diagnostic efficacy for death in the 60 to 90 age group. (B) Diagnostic efficacy for death in the 60 to 75 age group. (C) Diagnostic efficacy for death in the 75 to 90 age group. (D) Diagnostic efficacy for sepsis-related acute kidney injury in the 60 to 90 age group. (E) Diagnostic efficacy for sepsis-related acute kidney injury in the 60 to 75 age group. (F) Diagnostic efficacy for sepsis-related acute kidney injury in the 75 to 90 age group. *P*-values were calculated by log-rank test. AKI = acute kidney injury, ROC = receiver operating characteristic.

In the preliminary logistic regression analysis, we included variables that showed statistically significant differences (*P* < .05) as well as those with borderline significance (*P* < .10) to ensure that clinically relevant predictors were not excluded. Composite predictors were then derived by weighting the individual variables based on their regression coefficients (β values) within each age group. ROC curve analysis was used to evaluate the discriminative capacity of both individual and combined predictors of mortality outcomes. As summarized in Table 4 and Fig.2, distinct age-related patterns in the predictive performance were observed.

**Table 4:** ROC curve analysis of positive variables for logistic regression of death outcomes and survival analysis based on the best cutoff value between different age groups.

Variables	*P*-value*	AUC	95% CI low	95% CI high	Cutoff value	Sensitivity	Specificity	*P*-value†
Age 60–90								
LDH	<.001	0.565	0.55	0.58	4.55	0.863	0.246	
Urea nitrogen	<.001	0.588	0.57	0.60	26.5	0.496	0.643	
Creatinine	<.001	0.558	0.54	0.57	1.65	0.668	0.442	
Urine output	<.001	0.614	0.60	0.63	1046.5	0.68	0.508	
Hemoglobin	<.001	0.515	0.50	0.53	10.55	0.728	0.303	
Combination	<.001	0.631	0.62	0.64	11.908	0.741	0.45	<.001
Age 60–74								
LDH	<.001	0.605	0.58	0.63	2.65	0.646	0.513	
Urea nitrogen	<.001	0.579	0.56	0.60	27.5	0.513	0.617	
Creatinine	.001	0.561	0.54	0.58	1.45	0.564	0.565	
Urine output	<.001	0.635	0.62	0.66	1047.5	0.709	0.51	
P/F ratio	.008	0.557	0.53	0.58	150.5	0.665	0.444	
Combination	<.001	0.645	0.63	0.66	0.309	0.691	0.528	<.001
Age 75–90								
LDH	<.001	0.526	0.50	0.55	5.17	0.912	0.174	
Urea nitrogen	<.001	0.597	0.58	0.62	31.5	0.607	0.544	<.001
Urine output	.054	0.59	0.57	0.61	1147	0.6	0.556	
Albumin	<.001	0.576	0.55	0.61	2.65	0.726	0.386	
Hemoglobin	<.001	0.518	0.50	0.54	8.15	0.295	0.742	
Combination	.007	0.512	0.49	0.53	20.141	0.826	0.226	

AUC = area under the curve, CI = confidence interval, LDH = lactate dehydrogenase ROC = receiver operating characteristic.

* The *P*-value for logistic regression between variables and death outcomes.

† The *P*-value for survival analyses conducted based on grouping of the best cutoff values for variables with the largest AUC values.

For the 60 to 90 years cohort, LDH (area under the curve, AUC: 0.588), urea nitrogen (AUC: 0.604), creatinine (AUC: 0.515), urine output (AUC: 0.631), and hemoglobin (AUC: 0.527) all demonstrated significant predictive values for mortality. The composite predictor, which integrated these variables, achieved an AUC of 0.631 (95% CI: 0.612–0.650), indicating a moderate discriminative ability.

In the 60 to 75 years subgroup, LDH level (AUC: 0.605) and urine output (AUC: 0.635) emerged as the most dominant predictors. The composite model for this group showed an improved AUC of 0.645 (95% CI: 0.621–0.669), highlighting its enhanced prognostic accuracy compared with individual markers.

In the 75 to 90 years subgroup, while urea nitrogen remained a relevant predictor (AUC: 0.597), the composite predictor displayed decreased performance (AUC: 0.512, 95% CI: 0.488–0.536), suggesting that conventional biomarkers may have diminished utility in advanced age populations.

However, urine output consistently demonstrated strong predictive value across all age groups, with AUC values ranging from 0.631 to 0.635. This underscores their clinical utility as reliable prognostic indicators of mortality. In contrast, hemoglobin showed paradoxical associations; elevated levels were associated with an increased mortality risk (OR: 1.14, *P* = .013) in the oldest subgroup, which may reflect hemoconcentration or compensatory erythropoiesis in the context of chronic illness.

### 3.4. Survival analysis

**Figure 3.**
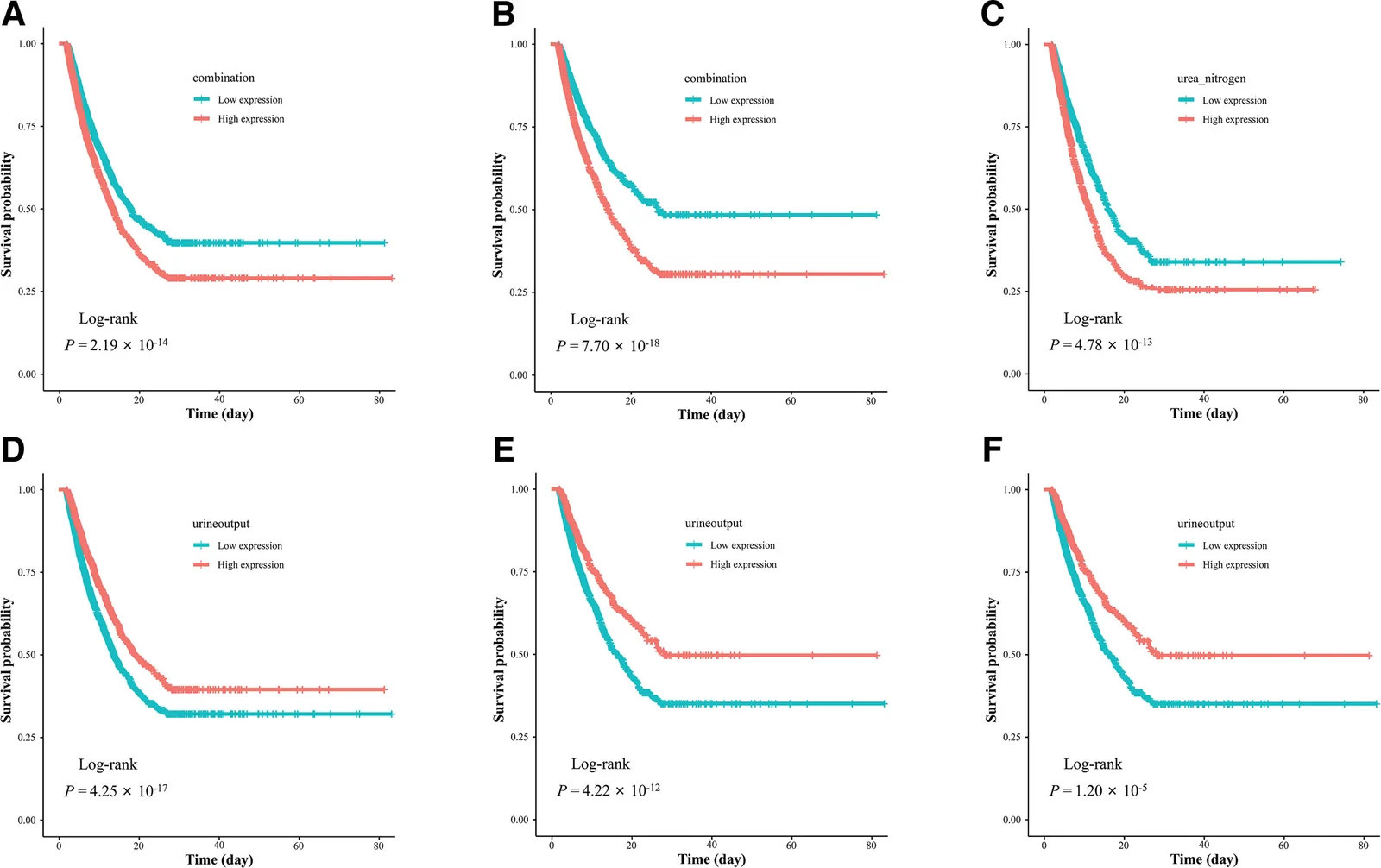
Survival analysis evaluating the impact of various predictor levels on different age groups. (A) Survival impact of combined predictor levels in the 60 to 90 age group. (B) Survival impact of combined predictor levels in the 60 to 75 age group. (C) Survival impact of urea nitrogen levels in the 75 to 90 age group. (D) Survival impact of urine output levels in the 60 to 90 age group. (E) Survival impact of urine output levels in the 60 to 75 age group. (F) Survival impact of urine output levels in the 75 to 90 age group.

Using ROC curve analysis, we identified the variables with the largest AUC in each age group and classified the study population into high- and low-expression groups based on their optimal cutoff values. Subsequently, we performed survival analysis using the Kaplan–Meier method to examine the relationship between the high and low expression groups and the outcome variables. The results indicated that the survival curves between the high- and low-expression groups were statistically significant across all age groups, as shown in Fig.3. The survival impacts of predictor levels varied across different age groups (60–90, 60–75, and 75–90). This suggests that age is a significant factor influencing survival outcomes and should be considered when interpreting the data. In panels (A) and (B), the analysis of combined predictor levels demonstrates significant differences in survival between high and low expression groups, particularly within the 60 to 90 and 60 to 75 age groups. This indicates that a combination of predictors may provide valuable insights into the survival probabilities of patients in these age categories, and panel (C) highlights the survival impact of urea nitrogen levels, specifically in the 75 to 90 age group. The significant difference in survival outcomes based on urea nitrogen levels suggests that this variable is a critical marker for assessing prognosis in older patients, particularly those in advanced age brackets. Panels (D), (E), and (F) consistently demonstrate that urine output significantly affects survival across all age groups analyzed. Notably, the strong significance in both the 60 to 90 and 75 to 90 age groups suggests that urine output is a crucial indicator of renal function and overall health status, potentially reflecting the severity of the underlying condition.

The consistent statistical significance (evidenced by low *P*-values from the log-rank tests) across all analyzed variables underscores the potential of these predictors (combined levels, urea nitrogen, and urine output) as effective prognostic indicators. They can aid clinicians in stratifying patients based on risk and in tailoring interventions accordingly. These findings emphasize the importance of regular monitoring of the identified predictors in clinical practice, particularly in older patients. Early identification of adverse prognostic indicators could lead to timely interventions, ultimately improving patient outcomes. The analysis revealed that various predictor levels significantly impact survival across different age groups, highlighting the importance of tailored prognostic assessments in clinical settings.

To further validate the association between the variables and the outcomes, we conducted a chi-square test on the distribution of the outcome variables between the high and low expression groups, as shown in Table 5. The results demonstrated statistically significant differences in the distribution of these variables between groups. Finally, for the same expression level group (either high or low expression), we compared the distribution of different outcome variables using independent sample *t*-tests, revealing significant statistical differences in the distribution of variables among the groups.

**Table 5:** The chi-square test between high and low expression groups and outcome variables.

Variables	Death		*χ* ^2^	*P*-value	Sepsis AKI		*χ* ^2^	*P*-value
	0	1			1	2		
Age 60–90								
Combination								
Low	5234	1240	294.782					
High	1826	1014						
Urine output								
Low					330	2641	652.288	<.001
High					1263	1924		
Age 60–75								
Combination								
Low	2512	483	166.977	<.001				
High	1122	540						
Urine output								
Low					193	1349	320.970	<.001
High					589	827		
Age 75–90								
Urea nitrogen								
Low	2073	559	83.869	<.001				
High	1342	667						
Urine output								
Low					156	1386	364.706	<.001
High					655	1003		

AKI = acute kidney injury.

## 4. Discussion

Our analysis of age-stratified elderly sepsis cohorts from the MIMIC-IV database revealed critical insights into the dynamic interplay between aging physiology and sepsis prognosis. Three central themes emerged from the findings: the shifting hierarchy of prognostic biomarkers across age subgroups, the paradoxical utility of composite predictors in younger versus older elderly patients, and the universal prognostic dominance of urine output as a clinical sentinel. These patterns challenge conventional “one-size-fits-all” approaches to sepsis prognostication and underscore the importance of age-tailored risk-stratification models.

The differential predictive performance of biomarkers between the younger (60–74 years) and older elderly (75–90 years) cohorts underscores the biological complexity of aging in sepsis. LDH, a marker of cellular injury, demonstrated a stronger prognostic value in younger elderly patients (AUC, 0.605 vs 0.526), potentially reflecting their preserved capacity for acute-phase metabolic responses.^[9,10]^ In contrast, urea nitrogen emerged as the dominant predictor in older elderly patients (AUC, 0.597), likely mirroring the cumulative burden of age-related renal senescence and reduced glomerular reserve. This transition from cellular injury markers to renal function indicators aligns with longitudinal studies documenting progressive nephron loss and altered drug metabolism in advanced aging populations.^[11,12]^ Notably, the protective association of albumin in older patients (OR 0.70, *P* = .048) may reflect its dual role as both a nutritional marker and modulator of endothelial integrity, a finding consistent with malnutrition-inflammation complex syndromes prevalent in geriatric critical care.^[13]^

While biomarker integration enhanced prognostic accuracy in younger elderly patients (AUC 0.645), its diminished utility in the oldest subgroup (AUC 0.512) revealed the fundamental limitations of current prognostic paradigms. This divergence suggests that traditional biochemical markers fail to capture the multidimensional frailty determinants of cognitive decline, polypharmacy, and functional dependence, which increasingly dominate outcomes in advanced age.^[14]^ The observed hemoglobin paradox, in which elevated levels predicted mortality in older patients (OR 1.14, *P* = .013), exemplifies this complexity. Rather than indicating true erythrocytosis, this association may signal hemoconcentration from chronic dehydration or compensatory erythropoiesis in chronic disease states, phenomena rarely quantified in routine sepsis assessments.

Across all age strata, urine output maintained a robust predictive value (AUC 0.631–0.635), transcending its traditional role as an AKI marker. In elderly patients with sepsis, oliguria is likely to represent a composite signal of systemic hypoperfusion, endothelial dysfunction, and neurohormonal dysregulation. The escalating prognostic weight with age, particularly in 75 to 90 to year-olds, may reflect diminishing renal adaptive capacity, where even subclinical perfusion deficits precipitate metabolic cascades. This aligns with microcirculatory studies showing age-related impairments in renal autoregulation that predispose patients to ischemic injury despite preserved macrovascular flow.^[15]^ Clinically, these findings reinforce the role of urine output as a prophylactic in coal mines “ for early intervention, particularly given its cost-effectiveness and real-time measurability.

The striking mortality gradient revealed by Kaplan–Meier analyses, particularly the 2.5-fold risk increase with urea nitrogen >35 mg/dL in older patients, demands urgent clinical attention. These thresholds could inform dynamic treatment algorithms such as early nephrotoxin avoidance protocols for elderly patients with azotemia. Furthermore, the temporal stability of the predictive power of urine output suggests its potential as a triage tool in resource-limited settings. However, the diminishing returns of composite models in advanced age groups highlight the need to integrate nontraditional variables-functional status, caregiver support, and advance care preferences-into prognostic frameworks.^[16-18]^ The observed biomarker patterns may reflect fundamental aging processes. Elevated LDH levels in younger elderly patients could signify heightened cellular stress responses in relatively preserved tissues, whereas chronic subclinical organ injury in older patients may blunt acute-phase biomarker elevations. The renal prognostic shift mirrors autopsy studies demonstrating 30% to 50% nephron loss by age 80, creating a precarious balance between metabolic demand and excretory capacity.^[19,20]^ Concurrently, the association between hypoalbuminemia and mortality in older patients exposes the vicious cycle of chronic inflammation and protein catabolism, a therapeutic target for nutritional interventions in sepsis protocols.^[21]^

Our findings have 3 practical implications. First, age-stratified sepsis bundles should prioritize LDH-driven organ support in younger elderly patients versus renal-protective strategies in older cohorts. Second, dynamic biomarker monitoring, particularly serial urine output and urea nitrogen trending, could enable the earlier detection of clinical deterioration. Third, prognostic models must evolve to incorporate geriatric syndromes, potentially through frailty indices or sarcopenia assessment. Future research should validate these findings in multicenter cohorts while exploring multi-omics approaches to identify novel age-specific biomarkers. Several constraints contextualize our findings. The retrospective design precludes causal inferences, whereas single-center data may limit generalizability. First-day biomarker sampling potentially misses evolving prognostic patterns, and the exclusion of nonagenarians leaves open questions regarding extreme-age sepsis biology. Prospective studies integrating functional assessments and biomarker trajectories across care settings are required to address these gaps.

## 5. Conclusion

This study delineates the critical role of age-specific prognostic factors, LDH, urea nitrogen, and urine output, in elderly patients with sepsis mortality. By transcending a “one-size-fits-all” approach and embracing age-stratified care, clinicians can enhance risk prediction, personalize interventions, and ultimately improve outcomes in this vulnerable population. Our findings call for reimagining sepsis management paradigms to reflect the biological and clinical complexity of aging.

## Author contributions

**Data curation:** Jiao Chen, Jinfeng Lin.

**Formal analysis:** Jiao Chen, Jinfeng Lin, Chunfeng Gu.

**Funding acquisition:** Jiao Chen.

**Investigation:** Jiao Chen, Jinfeng Lin, Hao Wang.

**Methodology:** Jiao Chen, Jinfeng Lin.

**Project administration:** Jiao Chen, Chunfeng Gu, Xiang Lu, Chongxiu Sun.

**Software:** Jinfeng Lin, Chunfeng Gu.

**Supervision:** Hao Wang.

**Validation:** Hao Wang.

**Visualization:** Chongxiu Sun.

**Writing – review & editing:** Xiang Lu, Chongxiu Sun.

**Writing – original draft:** Jiao Chen
